# Brazilian National Curricular Guidelines for Undergraduate Nursing: ABEn in defense of education and SUS as public goods

**DOI:** 10.1590/0034-7167.2025780102

**Published:** 2025-03-17

**Authors:** Célia Alves Rozendo, Adriana Katia Corrêa, Dagmar Elaine Kaiser, Jacinta de Fátima Sena da Silva, José Renato Gatto, Josicelia Dumêt Fernandes, Kênia Lara da Silva

**Affiliations:** I Universidade Federal de Alagoas. Maceió, Alagoas, Brazil; II Universidade de São Paulo, Escola de Enfermagem de Ribeirão Preto. Ribeirão Preto, São Paulo, Brazil; III Universidade Federal do Rio Grande do Sul, Escola de Enfermagem e Saúde Coletiva. Porto Alegre, Rio Grande do Sul, Brazil; IV Universidade de Brasília, Departamento de Saúde Coletiva. Brasília, Distrito Federal, Brazil.; V Universidade de São Paulo, Escola de Enfermagem de Ribeirão Preto. Ribeirão Preto, São Paulo, Brazil; VI Universidade Federal da Bahia, Salvador, Bahia, Brazil; VII Universidade Federal de Minas Gerais, Escola de Enfermagem. Belo Horizonte, Minas Gerais, Brazil

The first Brazilian National Curriculum Guidelines for Undergraduate Nursing (In Portuguese, *Diretrizes Curriculares Nacionais da Graduação em Enfermagem* - DCN/ENF) were enacted 24 years ago. Over this period, political, economic, social, cultural, technological, and health changes, among others, gradually pointed to the imperative need for review and updating, with the proposal of new DCN/ENF, a movement led by the Brazilian Nursing Association (In Portuguese, *Associação Brasileira de Enfermagem* - ABEn) together with Brazilian nursing.

In the period between the DCN/ENF, approved in 2001, and the process of their updating, there was an expansion of neoliberal policies, inserted in the context of strengthening financial capital, on a global scale, which greatly impacted the privatization and commercialization of higher education in Brazil. One of the manifestations of this phenomenon has been the vertiginous and uncontrolled growth of distance learning^(1)^. Undergraduate nursing education stands out in this scenario, with implications for training that compromise conceptual, theoretical, and practical soundness as well as critical thinking and social and ethical commitment. Data from the 2022 Higher Education Census express the gravity of the current situation mentioned above, indicating the offer of 4,062 undergraduate nursing courses in Brazil, of which 2,759 are in the distance learning modality, accounting for 67% of the total.

In the health field, recent years have been marked by struggles against privatization, in defense of the Brazilian Health System (In Portuguese, *Sistema Único de Saúde* - SUS) and the guarantee of health as a citizen’s right and a State’s duty^(2)^. Changes have also been observed in the world of work, including labor and social security reforms, which represent a setback in the rights achieved by workers.

One event that deserves to be highlighted was the COVID-19 pandemic, which broke out in 2020, which further highlighted social inequalities, bringing implications for the training of health workers. The incorporation of teaching technologies, while on the one hand, made it possible to address the limits imposed by the need for social isolation, on the other hand, led to their uncritical incorporation instead of considering the inequalities of Brazilian students and the purposes of training^(3)^.

Moreover, important social movements have also been marking the last decade, considering ethnic-racial and gender relations, human rights, environmental education and sustainability, bringing to light the distinct diversities in the Brazilian political, social and cultural context and, along with them, the countless challenges, including that of guaranteeing life on the planet.

In this complex and diverse scenario, during all ABEn administrations after 2012, efforts were made to develop new DCN/ENF. The development process was carried out in a collective and democratic manner, seeking to bring nursing together around an education project that, in fact, faces changes with criticism, ethics and social responsibility, and produces responses that positively impact citizens’ lives.

After successive back and forth of documents between ABEn and the Brazilian National Education Council/Chamber of Higher Education (In Portuguese, *Conselho Nacional de Educação/Câmara de Educação Superior* - CNE/CES), Ministry of Education, between 2017 and 2024, marked by many dialogues and negotiations, it can be stated that there were significant advances in the final document, presented in Opinion CNE/CES 443/2024, approved on July 3, 2024^(4)^, currently awaiting approval by the Minister of Education.

Some of these advances, with regard to bachelor’s degree, can be summarized as follows: maintenance of the inseparable articulation between undergraduate nursing courses and SUS, recognized as a public policy and a project for society; emphasis on care as the purpose of nurses’ work process; and reinforcement of generalist training with reference to professional areas that include care, management of care and services, professional development, research and health education. Also noteworthy is the definition of theoretical, theoretical-practical activities and mandatory supervised curricular internship, with indications of workloads and the student/professor/preceptor quantitative ratio.

Another important development concerns nursing degree, which now occupies a specific chapter in the DCN text. There is the incorporation of principles and knowledge that highlight the purpose of training nursing professors for teaching in secondary-level technical professional education, a basic education modality in which technicians are trained, whose actions have an impact on SUS.

It is expected that the new DCN/ENF will be a device that enables the technical-scientific and ethical-political training of workers committed to the fight for better living conditions for the population, with the maintenance of health and education as rights and, therefore, with the defense of SUS and of quality and socially referenced public education.

To this end, ABEn remains engaged and involved in the struggles that are of interest to nursing and the Brazilian people, fulfilling its important political, articulatory and dialogic mission to guarantee the prerogative of SUS as the organizer of the training of health workers. In this regard, it remains committed and making efforts to approve the new DCN/ENF, aware and attentive to the movements that permeate the current political context, understanding, above all, that it is necessary to have hope.
