# Supplementary material for: Brazilian National Curricular Guidelines for Undergraduate Nursing: ABEn in defense of education and SUS as public goods
Source: Rev Bras Enferm. 2025 Mar 17;78(1):e780102. doi: 10.1590/0034-7167.2025780102 (PMC11913425; doi:10.1590/0034-7167.2025780102)
Supplement: Translation

# Translation: Diretrizes Curriculares Nacionais da Graduação em Enfermagem: ABEn na defesa da educação e do SUS como bens públicos

**DOI:** 10.1590/0034-7167.2025780102pt

**Authors:** Célia Alves Rozendo, Adriana Katia Corrêa, Dagmar Elaine Kaiser, Jacinta de Fátima Sena da Silva, José Renato Gatto, Josicelia Dumêt Fernandes, Kênia Lara da Silva

**Affiliations:** I Universidade Federal de Alagoas. Maceió, Alagoas, Brasil; II Universidade de São Paulo, Escola de Enfermagem de Ribeirão Preto. Ribeirão Preto, São Paulo, Brasil; III Universidade Federal do Rio Grande do Sul, Escola de Enfermagem e Saúde Coletiva. Porto Alegre, Rio Grande do Sul, Brasil; IV Universidade de Brasília, Departamento de Saúde Coletiva. Brasília, Distrito Federal, Brasil.; V Universidade de São Paulo, Escola de Enfermagem de Ribeirão Preto. Ribeirão Preto, São Paulo, Brasil; VI Universidade Federal da Bahia, Salvador, Bahia, Brasil; VII Universidade Federal de Minas Gerais, Escola de Enfermagem. Belo Horizonte, Minas Gerais, Brasil

As primeiras Diretrizes Curriculares Nacionais da Graduação em Enfermagem (DCN/ENF) vigentes foram promulgadas há 24 anos. Ao longo desse período, mudanças políticas, econômicas, sociais, culturais, tecnológicas, sanitárias, entre outras, foram gradualmente apontando para a imperiosidade de revisão e atualização, com proposição de novas DCN/ENF, movimento liderado pela Associação Brasileira de Enfermagem (ABEn) em conjunto com a enfermagem brasileira.

No período decorrido entre as DCN/ENF, aprovadas em 2001, e o processo de sua atualização, observou-se a expansão das políticas neoliberais, inseridas no contexto de fortalecimento do capital financeiro, em âmbito mundial, que impactaram sobremaneira a privatização e mercantilização da educação superior no Brasil. Uma das manifestações desse fenômeno tem sido o crescimento vertiginoso e descontrolado do ensino à distância^(1)^. O ensino de graduação em enfermagem se destaca nesse cenário, com implicações na formação que comprometem a solidez conceitual, teórica, prática, além da criticidade e do compromisso social e ético. Dados do Censo da Educação Superior de 2022 expressam a gravidade da atual situação anteriormente referida, apontando a oferta de 4.062 cursos de graduação em enfermagem no Brasil, dos quais 2.759 são na modalidade à distância, representando 67% do total.

No campo da saúde, os últimos anos foram marcados por lutas contra a lógica privatista, em defesa do Sistema Único de Saúde (SUS) e da garantia da saúde como direito do cidadão e dever do Estado^(2)^. Vêm sendo observadas, também, mudanças no mundo do trabalho, incluindo as reformas trabalhista e da previdência, que representam um retrocesso nos direitos conquistados pelos trabalhadores.

Um acontecimento que merece destaque foi a pandemia de COVID-19, deflagrada em 2020, que evidenciou ainda mais as desigualdades sociais, trazendo implicações para a formação de trabalhadores da área da saúde. A incorporação de tecnologias de ensino, se, por um lado, possibilitou o enfrentamento dos limites impostos pela necessidade do isolamento social, por outro, levou à sua incorporação acrítica, em detrimento da consideração das desigualdades dos estudantes brasileiros e das finalidades da formação^(3)^.

Além disso, importantes movimentos sociais também vêm marcando a última década, considerando as relações étnico-raciais e de gênero, os direitos humanos, a educação ambiental e a sustentabilidade, trazendo à tona as distintas diversidades no contexto político, social e cultural brasileiro e, junto com elas, os inúmeros desafios, incluindo o da garantia da vida no planeta.

Nesse complexo e diversificado cenário, durante todas as gestões da ABEn após 2012, empreenderam-se esforços para a construção de novas DCN/ENF. O processo de construção ocorreu de modo coletivo e democrático, na busca de agregar a enfermagem em torno de um projeto de educação que, de fato, enfrente as mudanças, com crítica, ética e responsabilidade social, e produza respostas que impactem positivamente a vida dos cidadãos.

Após sucessivas idas e vindas de documentos entre a ABEn e o Conselho Nacional de Educação/Câmara de Educação Superior (CNE/CES), Ministério da Educação, entre 2017 e 2024, marcadas por muitos diálogos e negociações, pode-se afirmar que houve avanços significativos no documento final, apresentado no Parecer CNE/CES nº 443/2024, aprovado em 3 de julho de 2024^(4)^, aguardando, no momento, homologação pelo Ministro da Educação.

Alguns desses avanços, no que tange à formação do bacharel, podem ser assim sintetizados: a manutenção da inseparável articulação entre a graduação em enfermagem e o SUS, reconhecido como política pública e projeto de sociedade; a ênfase no cuidado como a finalidade do processo de trabalho de enfermeiros; e o reforço da formação generalista com referência a áreas profissionais que contemplam o cuidado, a gestão do cuidado e dos serviços, o desenvolvimento profissional, a pesquisa e a educação em saúde. Destaca-se, ainda, a definição de atividades teóricas, teórico-práticas e de estágio curricular supervisionado obrigatório, com indicativos de cargas horárias e da relação quantitativa estudante/professor/preceptor.

Outro avanço a ser destacado diz respeito à licenciatura em enfermagem, que passa a ocupar um capítulo específico no texto das DCN. Há a incorporação de princípios e conhecimentos que destacam a finalidade da formação de professores enfermeiros para a docência na educação profissional técnica de nível médio, modalidade da educação básica, na qual são formados os técnicos, cujas ações são impactantes no SUS.

Espera-se que as novas DCN/ENF sejam um dispositivo que possibilite a formação técnico-científica e ético-política de trabalhadores comprometidos com a luta por melhores condições de vida da população, com a manutenção da saúde e da educação como direitos e, portanto, com a defesa do SUS e da educação pública de qualidade e socialmente referenciada.

Para tal, a ABEn segue participativa e engajada nas lutas que interessam à enfermagem e ao povo brasileiro, cumprindo sua importante missão política, articuladora e dialógica para garantir a prerrogativa do SUS como ordenador da formação de trabalhadores da área da saúde. Nesse sentido, mantém-se comprometida e engendrando esforços para a homologação das novas DCN/ENF, consciente e atenta aos movimentos que permeiam o atual contexto político, compreendendo, sobretudo, que é preciso ter esperança.

# Translation: Directrices Curriculares Nacionales de Graduación en Enfermería: ABEn en defensa de la educación y del SUS como bienes públicos

**DOI:** 10.1590/0034-7167.2025780102es

**Authors:** Célia Alves Rozendo, Adriana Katia Corrêa, Dagmar Elaine Kaiser, Jacinta de Fátima Sena da Silva, José Renato Gatto, Josicelia Dumêt Fernandes, Kênia Lara da Silva

**Affiliations:** I Universidade Federal de Alagoas. . Maceió, Alagoas, Brasil; II Universidade de São Paulo, Escola de Enfermagem de Ribeirão Preto. Ribeirão Preto, São Paulo, Brasil; III Universidade Federal do Rio Grande do Sul, Escola de Enfermagem e Saúde Coletiva. Porto Alegre, Rio Grande do Sul, Brasil; IV Universidade de Brasília, Departamento de Saúde Coletiva. Brasília, Distrito Federal, Brasil.; V Universidade de São Paulo, Escola de Enfermagem de Ribeirão Preto. Ribeirão Preto, São Paulo, Brasil; VI Universidade Federal da Bahia, Professora Emérita da Escola de Enfermagem. Salvador, Bahia, Brasil; VII Universidade Federal de Minas Gerais, Escola de Enfermagem. Belo Horizonte, Minas Gerais, Brasil

Hace 24 años se promulgaron las primeras Directrices Curriculares Nacionales de Graduación en Enfermería (En portugués, *Diretrizes Curriculares Nacionais da Graduação em Enfermagem* - DCN/ENF) vigentes. A lo largo de este período, los cambios políticos, económicos, sociales, culturales, tecnológicos, de salud, entre otros, apuntaron gradualmente al imperativo de revisión y actualización, con la propuesta de la nueva DCN/ENF, movimiento liderado por la Asociación Brasileña de Enfermería (En portugués, *Associação Brasileira de Enfermagem* - ABEn) junto con la enfermería brasileña.

En el período comprendido entre las DCN/ENF, aprobadas en 2001, y el proceso de actualización de las mismas, se observó la expansión de las políticas neoliberales, insertas en el contexto de fortalecimiento del capital financiero, a nivel mundial, lo que impactó grandemente en la privatización y mercantilización de la educación superior en Brasil. Una de las manifestaciones de este fenómeno ha sido el crecimiento vertiginoso y descontrolado de la educación a distancia^(1)^. En este escenario se destaca la formación de pregrado en enfermería, con implicaciones para la formación que comprometen la solidez conceptual, teórica y práctica, así como la criticidad y el compromiso social y ético. Los datos del Censo de Educación Superior de 2022 expresan la gravedad de la situación actual mencionada anteriormente, señalando la oferta de 4.062 cursos de pregrado en enfermería en Brasil, de los cuales 2.759 son a distancia, lo que representa el 67% del total.

En el campo de la salud, los últimos años han estado marcados por luchas contra la lógica privatista, en defensa del Sistema Único de Salud (En portugués, *Sistema Único de Saúde* - SUS) y de la garantía de la salud como derecho de los ciudadanos y deber del Estado^(2)^. También se han observado cambios en el mundo del trabajo, incluidas reformas laborales y de seguridad social, que suponen un retroceso en los derechos alcanzados por los trabajadores.

Un evento que merece ser destacado fue la pandemia de COVID-19, desencadenada en 2020, que puso de relieve aún más las desigualdades sociales, trayendo implicaciones para la formación de los trabajadores de la salud. La incorporación de tecnologías de enseñanza, si, por un lado, permitió enfrentar los límites impuestos por la necesidad de aislamiento social, por el otro, condujo a su incorporación acrítica, en detrimento de considerar las desigualdades de los estudiantes brasileños y los propósitos de la formación^(3)^.

Además, importantes movimientos sociales también marcaron la última década, considerando las relaciones étnico-raciales y de género, los derechos humanos, la educación ambiental y la sostenibilidad, sacando a la luz las distintas diversidades del contexto político, social y cultural brasileño y, con ellas, los innumerables desafíos, incluido el de garantizar la vida en el planeta.

En este escenario complejo y diverso, durante todas las administraciones de ABEn posteriores a 2012, se realizaron esfuerzos para construir nuevas DCN/ENF. El proceso de construcción se desarrolló de manera colectiva y democrática, en la búsqueda de agregar la enfermería en torno a un proyecto educativo que, efectivamente, afronte los cambios, con crítica, ética y responsabilidad social, y produzca respuestas que impacten positivamente en la vida de los ciudadanos.

Después de sucesivos idas y venidas de documentos entre la ABEn y el Consejo Nacional de Educación/Cámara de Educación Superior (En portugués, *Conselho Nacional de Educação/Câmara de Educação Superior* - CNE/CES), Ministerio de Educación, entre 2017 y 2024, marcados por muchos diálogos y negociaciones, se puede decir que hubo avances significativos en el documento final, presentado en el Dictamen CNE/CES nº 443/2024, aprobado el 3 de julio de 2024^(4)^, actualmente pendiente de aprobación por parte del Ministro de Educación.

Algunos de estos avances, en lo que respecta a la formación de licenciatura, se pueden resumir en los siguientes: el mantenimiento de la articulación inseparable entre la graduación en enfermería y el SUS, reconocido como una política pública y un proyecto de sociedad; el énfasis en el cuidado como finalidad del proceso de trabajo del enfermero; y fortalecer la formación generalista con referencia a áreas profesionales que incluyen la atención, la gestión asistencial y de servicios, el desarrollo profesional, la investigación y la educación sanitaria. Destaca también la definición de actividades teóricas, teórico-prácticas y prácticas curriculares tuteladas obligatorias, con cargas horarias indicativas y la relación cuantitativa estudiante/docente/preceptor.

Otro avance a destacar se refiere al grado en enfermería, que ahora ocupa un capítulo específico en el texto de la DCN. Se incorpora principios y conocimientos que resaltan el propósito de formar enfermeros docentes para la docencia en la educación profesional técnica secundaria, modalidad de educación básica, en la que se forman técnicos, cuyas acciones tienen impacto en el SUS.

Se espera que el nuevo DCN/ENF sea un dispositivo que permita la formación técnico-científica y ético-política de trabajadores comprometidos con la lucha por mejores condiciones de vida de la población, con el mantenimiento de la salud y la educación como derechos y, por tanto, con la defensa del SUS y de una educación pública de calidad y referenciada socialmente.

Para ello, la ABEn permanece participativa y comprometida en las luchas que interesan a la enfermería y al pueblo brasileño, cumpliendo su importante misión política, articuladora y dialógica de garantizar la prerrogativa del SUS como organizador de la formación de los trabajadores de la salud. En este sentido, mantiene su compromiso y se esfuerza por aprobar la nueva DCN/ENF, consciente y atenta a los movimientos que permean el contexto político actual, entendiendo, sobre todo, que es necesario tener esperanza.
